# The combination of methotrexate and cytosine arabinoside in newly diagnosed adult Langerhans cell histiocytosis: a prospective phase II interventional clinical trial

**DOI:** 10.1186/s12885-020-06872-8

**Published:** 2020-05-18

**Authors:** Xiao Han, Mingqi Ouyang, Minghui Duan, Wei Zhang, Tienan Zhu, Jian Li, Shujie Wang, Daobin Zhou

**Affiliations:** Department of Hematology, Peking Union Medical College Hospital, Chinese Academy of Medical Sciences and Peking Union Medical College, No.1 Shuaifuyuan, Beijing, People’s Republic of China

**Keywords:** Langerhans cell histiocytosis, Methotrexate, Cytosine arabinoside, Efficacy, Toxicity

## Abstract

**Background:**

Langerhans Cell Histiocytosis (LCH) is a rare disease puzzling both children and adults, however outcome of adult patients is unfavorable. This prospective interventional trial aims to test the efficacy and safety of the combination of methotrexate and cytosine arabinoside in adult LCH patients.

**Method:**

A total of 36 patients enrolled diagnosed with LCH and treated in our center from 1st Jan, 2014 to 30th Jun, 2016.

**Result:**

Nineteen patients underwent the detection of *BRAF* mutation, with a positive rate of 21.1%. The overall response rate was 100%, only 16.7% achieved complete response. The overall regression rate of osseous lesions was 100%. Regression of central nervous system involvement was also favorable. After a median follow-up of 44 months, the estimated event-free survival was 48.9 months, the overall survival rate was 97.2%. The risk organ involvement showed strong prognostic value, EFS was 34.1 or 54.6 months (*p* = 0.001) in groups with/without risk organ involvement respectively. Neutropenia and thrombocytopenia were the most common adverse effects.

**Conclusion:**

The regimen of methotrexate and cytosine arabinoside (MA) is effective and safe in treating adult LCH patients, and timely preventions may be considered for the high incidence of hematological adverse effects.

**Trial registration:**

Trial No. NCT02389400 on Clinicaltrials.gov, registered on 10th Mar. 2015.

## Background

Langerhans Cell Histiocytosis (LCH) is a rare disease manifesting with broad spectrum in both children and adults, which seems to be predominant in children. The manifestations range from localized self-limiting lesions to life-threatening disseminated disease. The treatment decision is mainly based on the disease extent, and since the 1990s, the Histiocyte Society has conducted many randomized international trials to explore the effective and safe chemotherapy for LCH patients with multi-system involvement, and improve the outcomes [1–3]. They have achieved some results that significantly improved the outcomes of LCH patients, especially in patients with multi-system involvement. They suggest that pediatric Multiple System disease (MS) LCH patients is best managed with combination therapy for a prolonged duration, which may decrease the recurrence rate. However, the treatment for MS LCH patients is only well established in children, and most large-scale prospective clinical trial only involved pediatric patients as well [4, 5].

The data for treatment in adult LCH patients is limited to case report or case series with limited prospective clinical trial [6]. In general, the same diagnostic and therapeutic approaches are applied in adults as in children. The classical regimen consisting of vinblastine and prednisone is also applied in adult LCH patients, and cytarabine is recommended in adult patients with risk organ involvement or multifocal disease [6, 7]. However, according to our previous study, the VP (vindesine and prednisone) regimen showed limited efficacy in adults [8], and the prolongation of vinblastine may lead to irreversible neurotoxicity [9]. There are predicaments calling for other regimens set for adult LCH patients. Existing evidences have supported that cytarabine showed superior effects on osseous lesions, and was recommended for adult LCH patients in the literature [6, 10]. Cytarabine is a deoxycytidine analog that incorporates into DNA promoting strand breaks, they serve as alternative substrates for enzymes metabolizing naturally-occurring nucleosides and nucleotides and they compete with natural substrates. Cytarabine promotes both cell death and differentiation in leukemia cells and induces apoptosis through p38MAPK and JNK pathways [11, 12]. We also take lessons from our experience in treating primary central nervous system lymphoma (PCNSL) [13], and high dose intravenous methotrexate (MTX) is the most important and beneficial single agent in PCNSL [14–16], it acts as a potent inhibitor of dihydrofolate reductase (DHFR), and inhibition of thymidylate results in lack of DNA synthesis [17]. According to the current knowledge of LCH, central nervous system and osseous lesions accounted for most LCH patients, which drives us taking the specific effects on these two systems into account. Therefore, we proposed the combination of MTX and cytarabine as an effective regimen for newly diagnosed adult LCH patients. And we conducted this prospective interventional clinical trial, aiming to explore the efficacy and safety of this regimen. The dose of cytarabine was determined based on experiences reported in the literature, while dose of MTX was referred from the course B of hyperCVAD regimen [3, 13, 18].

## Method

### Study design and objectives

This trial (Trial No. NCT02389400) is a prospective, single-center, single-arm, phase 2 interventional clinical trial, the objective of the study was to explore the efficacy and toxicity of the combination chemotherapy of Methotrexate and cytosine arabinoside (MA) in newly diagnosed adult Langerhans Cell Histiocytosis.

### Patient entry and ethics approval

All patients enrolled in this trial had definitive pathological diagnosis of LCH, following the criteria defined by Histocyte Society (HS) [19], briefly described as morphologic identification of the characteristic LCH cells, positive staining of S100, CD1a and (or) Langerin (CD207), additional Birbeck granules in lesional cells by electron microscopy may be used for patients without CD207 staining. The eligible criteria include newly diagnosed patients with no prior treatment for LCH, and age from 18 to 60 years old. Patients with single-system lung involvement, pregnancy or lactation. The *BRAF-V600E* mutation was detected by real-time polymerase chain reaction using biopsy tissues.

The trial was approved by the Ethical Institutional Review Board of the Peking Union Medical College Hospital in accordance with the Declaration of Helsinki, and all patients have written informed consents.

### Disease extent

Extent of disease was confirmed with physical examination, laboratory and radiographic studies. The baseline evaluations were performed after the confirmed diagnosis by histopathology and before the first cycle of therapy. All patients were stratified as Multi-system at risk disease (MS-RO+), Multi-system low-risk disease (MS-RO-) and multifocal single-system disease (SS-m) [19, 20]. MS-RO+ was defined as multiple system disease with risk organ involvement, including liver, spleen, and bone marrow. The liver involvement was diagnosed by Hepatomegaly that exceeded 3 cm below costal margin, and/or liver dysfunction and/or liver biopsy, and splenic enlargement that exceeded 2 cm below the costal margin in the midclavicular line was defined as spleen involvement, while hemocytopenia in at least 2 linear (anemia, neutropenia, and thrombocytopenia) was used to determine the bone marrow involvement [19]. Single system disease included multifocal bone disease (defined as lesions in 2 or more sites), localized special site involvement, such as CNS-risk lesions with intracranial soft tissue extension or vertebral lesions with intraspinal soft tissue extension, and the CNS-risk lesions indicated lesion in temporal bone, mastoid, sphenoid bone, and orbital bone, but vault lesions were not regarded as CNS-risk lesions [19]. The Single-system multifocal group (SSm) was defined as single system LCH with multifocal bone lesions [19]. Patients diagnosed as multifocal single-system disease must undergo direct histopathological confirmation of lesions, and excluded the involvement of other organs. The bone involvement was diagnosed by direct pathological evidence and/or typical lytic bone lesions on baseline imaging results. The CNS involvement was confirmed by CNS symptoms (such as central diabetes insipidus, suspected endocrinal abnormality secondary to pituitary dysfunction, etc), with mass lesion on MRI of head or abnormal uptake on PET/CT. Furthermore, the lung involvement was diagnosed by typical changes on high-resolution CT scanning or histopathological evidence.

### Treatment protocol

The MA regimen consist of intravenous methotrexate (MTX, 1 g/m^2^ body surface area) on day 1 and intravenous cytosine arabinoside (0.1 g/m^2^ body surface area) on days 1 to 5 of every 28-day cycle. Taking the economic factors of most Chinese patients, as well as the reference from the therapy of lymphoma, the planned number of cycle was 6, unless disease progression. Patients confront Grade 4 adverse events may delay the chemotherapy. Conventional post-hydration protocol was employed after MTX infusion [21].

### Response evaluation

The status of LCH was classified into 4 types [19, 22], non-active disease (NAD), defined as complete resolution of all symptoms and signs, active disease (AD)-regressive, which means continuous regression of symptoms or signs without new lesions, AD-stable, defined as persistence of symptoms or signs without new lesions, and AD-progressive which means disease progression with or without new lesions. In accordance to the guideline by HS^19^, a favorable response to treatment was defined as response-Better, including NAD and AD-regressive, while response-Intermediate was defined as stable or mixed response, and response-Worse referred to disease progression.

The bone lesion response was evaluated by imaging scanning of bone lesions, such as PET-CT, MRI, or whole body bone scanning, we referred the RECIST criteria [23] in lymphoma as the criteria when evaluating the response of bone lesions, briefly the CR was defined as disappearance of all measurable lesions, PR was defined as decrease in target lesion diameter sum > 30%, PD means increase in target lesion diameter sum > 20%, and SD means lesions that cannot meet the criteria of other definitions [23]. In addition, the complaint of bone aching may be also taken into consideration after exclusion of other co-existing diseases that lead to the aching. And patients with bone aching was evaluated by their symptoms, the complete resolution was defined as complete relief of pain, while partial resolution means an obvious pain relief, progressive disease was defined as pain getting worse, while stable disease means change that cannot meet the criteria of CR, PR, or PD.

The CNS response was based on imaging scanning, and the criteria was referred from RANO criteria for high-grade glioma [24], briefly the CR was defined as complete disappearance of all enhanced lesions for at least 4 weeks, PR was defined as at least 50% decrease in the sum of perpendicular diameters of enhancing disease, PD was defined as at least 25% increase in the sum of perpendicular diameters of enhancing disease, while SD means changes not qualify for CR, PR, PD, but no new lesions observed. The imaging scanning.

The follow-up imaging scanning of bone lesions was performed in the first year after treatment, and only repeat when a new lesion or recurrences suspected. The scanning of CNS involvement was performed once yearly after treatment during follow-up. The recurrence was defined as the occurrence of both clinical manifestations and abnormalities on imaging or laboratory tests.

### Adverse effect

Toxicity or adverse events during chemotherapy were documented and classified by National Cancer Institute Common Terminology Criteria for Adverse Events (CTCAE) Version 3.0.

### Statistical analysis

Endpoints of the study include overall response rate (ORR) after the last cycle of therapy, overall survival, event-free survival (EFS), incidence of adverse effects, and the mortality or morbidity. The ORR is considered as the rate of non-active disease or active disease-regression; overall survival was calculated from the date of diagnosis until death or last follow-up, EFS was calculated from the date of diagnosis until date of event or the last follow-up. OS and EFS were estimated by Kaplan-Meier method. *P*-values < 0.05 were indicative of statistical significance, and Log-rank test was used. A power >80% required a sample number of 23 of the MA group to show the significant elongation of estimated EFS compared to our historical data from VP or CEVP group [8] (the historical estimated EFS was 12 months, the expected EFS is 30 months in the current study, 2-sided α = 5%, Stata 12.0, exponential test).

## Result

### Patient characteristics (Table 1)

**Fig. 1:**
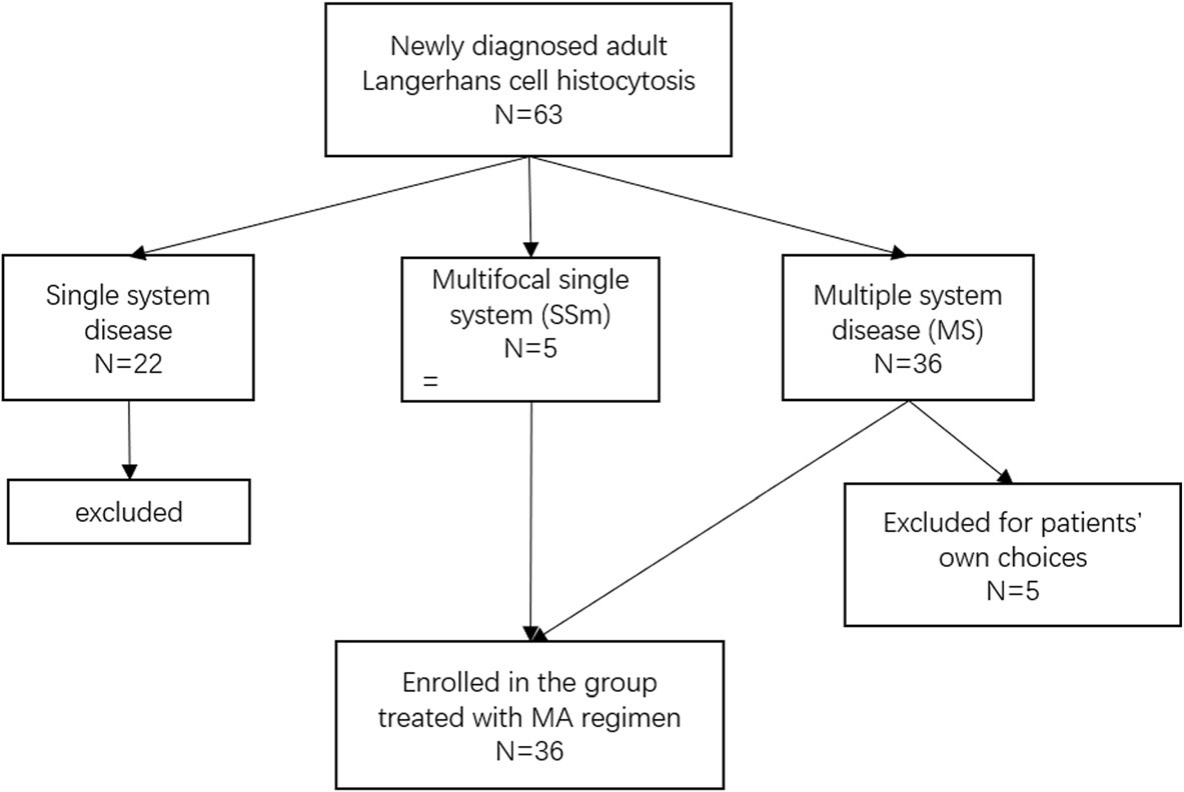
Patient flow in this trial with MA regimen

**Fig. 2:**
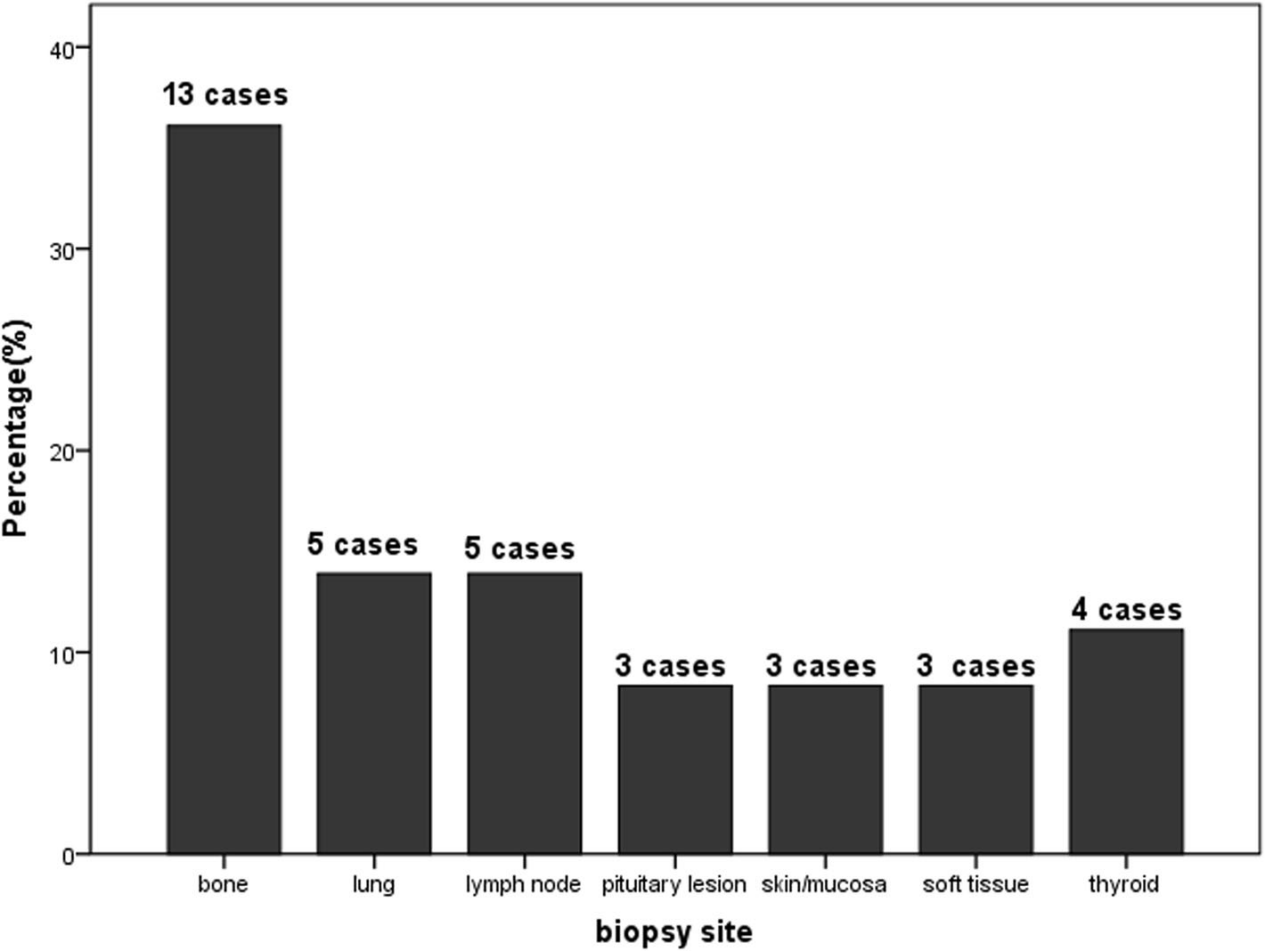
The biopsy site for definitive pathological diagnosis for patients enrolled

The enrollment started from Jan. 2014, and ended on Jun. 2016 as a medium-term report, the last follow-up ended on Dec. 2018. The patient flow was presented in Fig. 1, 63 cases of untreated adult LCH patients were newly diagnosed during the enrollment, including 22 cases of single system disease, who were not enrolled for not meeting the inclusion criteria, 5 cases with multifocal single system disease and 36 cases with multiple system disease. Five patients in MS group were excluded from the trial for rejection of MA regimen based on their own choices. A total of 36 patients were enrolled in the study. The mean age was 35.5 years old, with the range from 18 to 57 years old. The diagnostic pathology was obtained in all patients, and no patients had prior therapy. The origin of pathology included 13 cases of bone lesion (36.1%), 5 cases of both lung (13.9%) and lymph node (13.9%), 4 cases of thyroid (11.1%), and 3 cases of soft tissue (8.3%), skin/mucosa (8.3%) and pituitary lesion (8.3%) respectively (Fig. 2).

**Table 1:** Clinical characteristics of adult LCH patients at the time of diagnosis

	No.(ratio %)
Total patient	36
Gender	
Male	21 (58.3%)
Female	15 (41.7%)
Age at diagnosis (yrs)	
Median (range)	35.5 (18–57)
Disease classification	
Multifocal single system (SS-m)	5 (13.9%)
Multiple system without risk organ involvement (RO-)	20 (55.6%)
Multiple system with risk organ involvement (RO+)	11 (30.6%)
Liver involvement	10 (27.8%)
Spleen involvement	1 (2.8%)
Bone marrow (BM) involvement	1 (2.8%)
Other organ/system involvement	
Pulmonary involvement	21 (58.3%) 20/21 manifesting with pulmonary function disturbance.
Bone involvement	31 (86.1%)
Bone aching	18 (50%)
Lytic lesions	31 (86.1%)
Lesion sites	
Skull bone	22 (61.1%)
Vertebra	18 (50%)
Pelvis	13 (36.1%)
Ribs	11 (30.6%)
Extremity bones	8 (22.2%)
Central nervous system involvement	25 (69.4%)
Pituitary	23 (63.9%)
Other sites^a^	2 (5.6%)
Lymph nodes involvement	21 (58.3%)
CNS-risk organ involvement	8 (22.2%)
Thyroid involvement	7 (19.4%)
*BRAFV600E* mutation	
No. of patients tested	19
No. of positivity	4 (21.1%)

^a^other sites include hypothalamus in 1 cases and parenchyma near cranial basis in 1 case

A total of 5 patients had multifocal single system disease (SS-m), 20 patients had multiple system disease without risk organ involvement, and the remaining 11 patients had risk organ involvement. Among the patients who suffered from risk organ involvement disease, 10 patients had liver involvement, 1 had spleen involvement, while 1 patient had both liver and bone marrow involvement. All the 5 patients with SS-m disease showed multifocal bone involvement, and was confirmed by direct histopathological examinations. Bone was the most frequently involved organ in our research, counted for 31 patients, and all of them showed typical lytic bone lesions on imaging screening, while 18 of them complaint of bone aching, and the most common osseous site was skull bone. Secondary to bone involvement, central nervous system involvement (25 cases,69.4%) and lymph node enlargement (21 cases, 58.3%) were also quite common, central diabetes insipidus was the most common symptom (22 cases, 61.1%) for patients with CNS involvement, while the pituitary was the most common site involved (23 cases, 63.9%), the remaining 2 cases of CNS involvement included one case in hypothalamus and one case in parenchyma near cranial base revealed by MRI of head. Many patients had lung disease, showing typical changes on High-Resolution CT scanning, and manifested with lung function disturbance. The involvement of CNS-risk organ, skin/mucosa, and thyroid were also observed in the cohort. Table 1 shows the main clinical features of these 36 patients at diagnose.

A total number of 19 patients underwent the detection of *BRAF* V600E mutation, and 4 patients (21.1%) showed positive result validated by qPCR [25]. Among the 4 patients with positive BRAF V600E mutation, 2 belong to the SS-m group, and 2 belong to the MS-RO- group.

### Response and survival (Table 2)

#### Overall response

All patients had completed the planned 6 cycles of therapy. A total of 36 patients were available for response and survival analysis. After the completion of therapy, the ORR was 100%, 6 patients achieved NAD, while 30 patients achieved AD-regressive disease after MA treatment.

**Table 2:** Response to therapy and organ assessment

Cycles of chemotherapy	6
Patients available for survival analysis	36
Follow-up (mon)	44 (31–59)
EFS (mon)	39.5 (12–59)
Recurrence	10
Death	1 (death from disease progression)
Response (available for response evaluation)	36
Better	36
Complete resolution (NAD)	6
Regressive disease	30
Organ resolution	
Bone lesions (imaging evaluation)	31
CR	12
PR	19
Bone aching	18
Complete regression	14
Partial regression	4
CNS lesions (imaging evaluation)	25
CR	13
PR	12

#### Response for bone involvement

The response for bone was mainly evaluated by imaging scanning of bone lesions, 31 cases showed imaging abnormity on PET-CT, MRI, or whole-body bone scan, and 12 patients achieved CR with the disappearance of bone lesions, while 19 patients achieved PR showing obvious decrease of measurable bone lesions. In addition, the complaint of bone aching was greatly improved, showing that 14 patients achieved CR for the complete pain relief, while 4 patients achieved PR for an obvious pain relief after therapy.

In the SS-m group, 20% of patients achieved CR in osseous involvement, the remaining achieved PR. And in the MS-RO- group, 17 patients (85.0%) were diagnosed with bone involvement, showing that the rate of CR was 52.9% and the rate of PR was 47.1%. While in the MS-RO+ group, the rate of bone involvement was 81.8%, and the rate of CR and PR was 22.2 and 77.8% respectively.

#### Response for central nervous system involvement

A total of 25 patients showed CNS involvement, during the follow-up imaging scanning, 13 cases achieved CR, while 12 achieved PR on imaging results, and the ORR was 100%, patients with partial response on CNS involvement showed obvious shrinkage of pituitary lesions on MRI or reduced metabolic uptake on PET/CT, which are still abnormal. Although most of them had diabetes insipidus as long-term sequelae.

In the MS-RO- group, the rate of CNS involvement was 70%, while the rate of CR and PR was 64.3 and 35.7%. while in the MS-RO+ group, the CNS involvement occurred in all patients, showing the rate of CR and PR as 36.4 and 63.6% respectively.

### Survival and reactivation

**Fig. 3:**
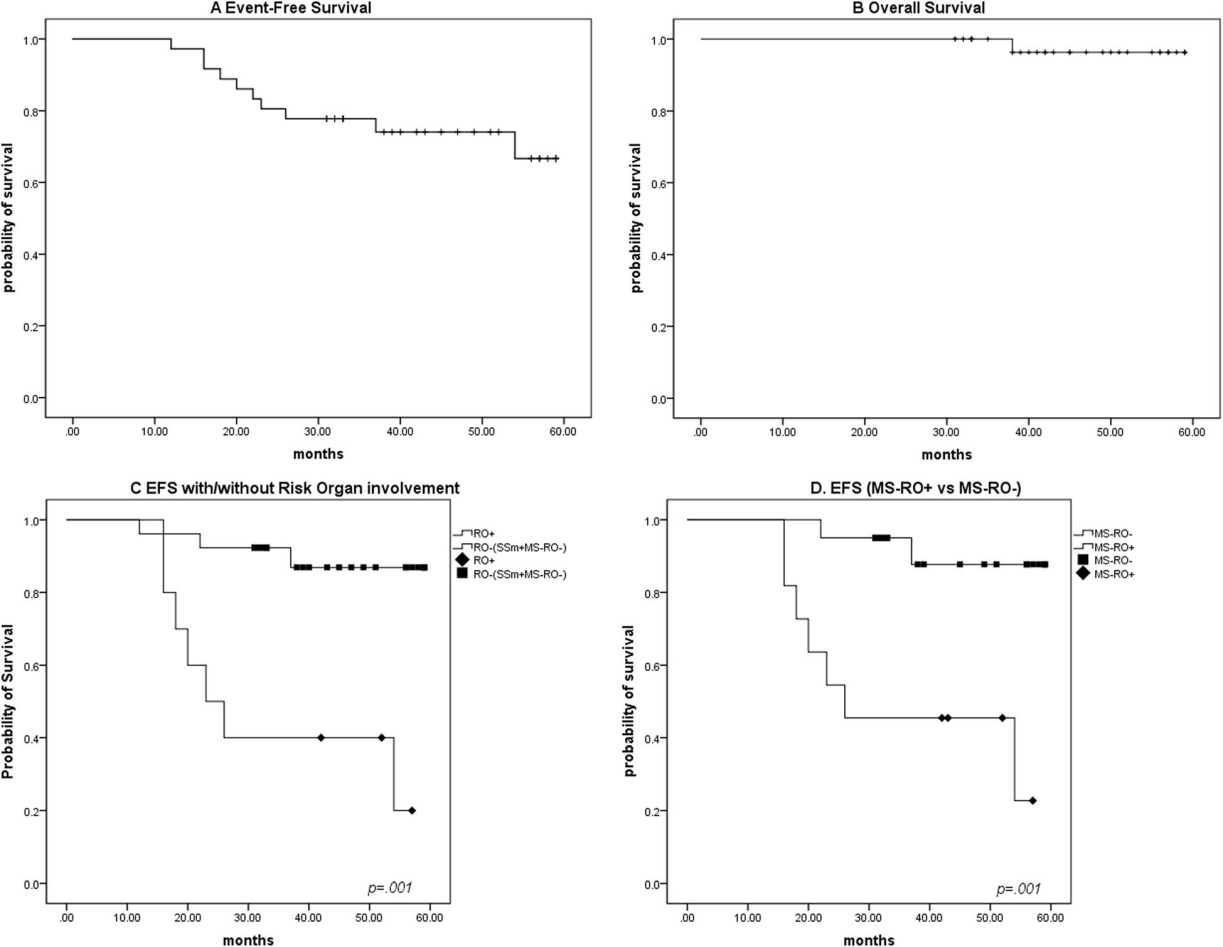
Survival of adult patients with LCH. **a** The overall EFS of the entire group in our study. **b** The overall survival of the entire group in our study. **c** The EFS of adult patients with and without risk organ involvement. The diamond represents group with risk organ involvement, while the square represents the group without risk organ involvement, including the multi-system disease and single system disease. **d** The EFS of adult patients with multi-system disease with and without risk organ involvement. The diamond represents MS-RO+ group, while the square represents the MS-RO- group

The median follow-up was 44 months, range 31–59 months. Among the whole cohort, 10 cases (27.8%) of reactivation were observed, 7 in MS-RO+ patients, and 2 in MS-RO- patients, while the remaining one patient in the SS group, the median time to relapse was 22 months (range: 12-54 months). One patient with MS-RO- disease died from disease progression quickly after recurrence, the interval between diagnosis and relapse was 35 months. The overall survival rate was 97.2% after a median follow-up of 44 months (1 death), the average EFS of this group was 48.9 months (95% CI: 43.4–54.5 months). The average EFS in patients with risk organ involvement was 34.1 months (95% CI: 23.1–45.1 months), which is significantly inferior to that in patients without risk organ involvement (average EFS: 54.6 months, 95% CI: 49.8–59.4 months, *p* = 0.001, Fig. 3), the latter group included patients with single-system disease and multi-system disease without risk organ involvement. Furthermore, The average EFS in MS-RO- group was 55.5 months (95% CI: 51.0–60.0 months), which is also significantly superior to that in MS-RO+ group (*p* = 0.001, Fig. 3). However, no significant difference was observed in other proposed groups (Supplement Figure S1).

### Adverse effects

No treatment-related death was observed in this study. Grade 4 adverse effects were only observed in hematological events, and neutropenia was the most common adverse effect observed. Among the 29 cases (80.6%) of neutropenia, the majority of patients manifested with grade 4 adverse effect (23 cases, 63.9%), while 5 cases (13.9%) with grade 3 neutropenia and 1 case (2.8%) with grade 2. Thrombocytopenia (15 cases, 41.7%) was quite common as well, grade 4 thrombocytopenia (platelet less than 25 × 10 [9]/L) was documented in 7 patients (19.4%). In addition to 2 cases of grade 3 infectious events (1 lung infection and 1 gastrointestinal infection), no more grade 3 or 4 non-hematological adverse effects were observed. Table 3 describes all adverse events of 36 patients during chemotherapy.

**Table 3:** Adverse effects in patients with LCH treated with MA regimen

Adverse Effects	No. (%)
Hematological toxicity	
Neutropenia	29 (80.6%)
Grade 2	1 (2.8%)
Grade 3	5 (13.9%)
Grade 4	23 (63.9%)
Thrombocytopenia	15 (41.7%)
Grade 1	1 (2.8%)
Grade 2	2 (5.6%)
Grade 3	5 (13.9%)
Grade 4	7 (19.4%)
Non-hematological toxicity	
Allergic reaction	8 (22.2%)
Grade 1	1 (2.8%)
Grade 2	7 (19.4%)
Liver function abnormality	3 (8.3%)
Grade 2	3 (8.3%)
Nausea	5 (13.9%)
Grade 1	1 (2.8%)
Grade 2	4 (11.1%)
Vomit	3 (8.3%)
Grade 1	2 (5.6%)
Grade 2	1 (2.8%)
Diarrhea	3 (8.3%)
Grade 1	3 (8.3%)
Infection event	4 (11.1%)
Grade 2	2 (1 case of gingival, and 1 case of skin)
Grade 3	2 (1 case of lung, and 1 case of digestive tract)

No death from chemotherapy observed

## Discussion

LCH is a disease that affect patients at all ages, but prominent in children with the estimated incidence of 3–5 per million patients [6, 26]. The precise incidence of LCH in adult is unknown but much lower than that in children. Studies focused on adult LCH are limited for its rarity, this current trial is one of the large prospective cohort in the literature of adult LCH. Unlike the slight female predominance reported by J. S. Malpas and A. J. Norton in 1996 [27], who also reported a large cohort in the literature, male patients counted more in our cohort. On the other hand, craniofacial osseous lesion is the most affected site in adult LCH patients which is similar to that in pediatric patients [28, 29], manifesting with lytic bone lesions. Central nervous system (CNS) is also one of the most affected site in LCH patients, and unfortunately, this involvement always leads to late sequelae that is hard to cure, and may bother patients through their lives. Central diabetes insipidus is the most common manifestation of CNS involvement [29], in this cohort the incidence of diabetes insipidus was 61.1%, which is much higher than the estimated rate of 25% [30]. The relative high incidence of diabetes insipidus may be resulted from the selection bias as our center is one of the largest general hospital and consultation center in China. In addition to hypothalamic pituitary region disease, neurodegenerative disease is also manifested as late sequelae with typical findings on magnetic resonance imaging (MRI), the median interval from diagnosis of this disease is 3.9 years according to previous study [31], but this was not evaluated in the cohort because of the limit of follow up.

The origin of LCH cell seems to be well understood as the deep insight of the molecular mechanism [32]. The detection of *BRAF* V600E active mutation has been widely employed in many studies [33, 34], and has lead the understanding of LCH to neoplastic nature. In addition to the *BRAF* mutation, somatic *MAP2K1* mutations also showed high prevalence in *BRAF* V600E–negative Langerhans cell histiocytosis [35], both *BRAF* and MAP2K1 mutation plays its role in mitogen-activated protein kinase (MAPK) activation pathway, supporting the central role of ERK activation in LCH pathogenesis [36, 37]. The reported rate of positivity of *BRAF* mutation is 40–65% [34, 36, 38, 39], in our group, only 21.1% patients showed positive *BRAF* mutation, the rate is lower than that in western studies, but is similar to a recent Japanese study, showing the rate of positive mutation of 20% [40]. In another Chinese group, the rate of positive mutation of BRAF is similarly low as 22.4% [41]. Although only 19 patients underwent *BRAF* mutation detection in our cohort, the similar lower result may speculate that there is a difference in the mutation between different races. Also, some studies have revealed that other somatic mutations of the MAPK pathway were recurrently detected in *BRAF* V600E-negative patients [35–37]. This result not only calls for the generalized application of *BRAF* mutation detection, also for the detection of other somatic mutations of the MAPK pathway.

One impressive result of our study is the high overall response rate of the combination of methotrexate and cytosine arabinoside. The treatment strategy is not satisfying in adult LCH patients, this situation leads to emergencies of many international studies that search for an effective and safe regimen, vinblastine combined with prednisone is the classical regimen used in pediatric LCH patients, showing favorable efficacy and safety [1, 4, 5]. Intensified treatment significantly increases rapid response and reduces mortality in risk MS-LCH, and the prolongation of duration did show some benefit [2, 3]. However, the resource in adult is limited, and these data showed relative lower efficacy and higher toxicity, and the prolongation of treatment seemed to increase the incidence of irreversible neurotoxicity [9]. Our previous study showed that the overall response rate of vindesine and prednisone (VP) regimen was 64.3%, and the intensive regimen of cyclophosphamide, etoposide, vindesine and prednisone (CEVP) was not superior to VP regimen, showing the overall response rate as 70% [8]. Another disadvantage of VP regimen in adult is the higher recurrence rate and mortality when compared to pediatric patients [28]. As a result, the unfavorable result has pushed forward other chemotherapy aiming to improve the prognosis of adult patients. When considering the combination of potential effective chemotherapy regimen, we paid special attention to the osseous response and CNS response because of the high incidence of involvement of these two systems. Cytosine arabinoside has achieved satisfying response when combined with other agents in pediatric patients, both the Japan LCH Study Group (JLSG) 96 protocol study and 02 protocol study [42, 43] reported appreciable results. In the JLSG-02 study, an intensified regimen containing cytosine arabinoside achieved good response or partial response rate (GR/PR) of 76.2% in RO+ group, and 93.7% in RO- group. Furthermore, in the study that focused on the bone lesions in adult LCH patients, which was reported by Cantu in 2012 [10], the results showed that single agent cytosine arabinoside achieved the best response when compared to other two chemotherapy regimen (VP regimen and single agent 2-CdA). The overall response rate in patients treated with Ara-C was 79%, which was much higher than that in other two groups, 16% in VP group and 41% in 2-CdA group. In terms of CNS involvement, Although MTX seems to show limited benefits in LCH-III study in children, high-dose MTX regimen still attracted our attention for its ability to get through blood-brain barrier [14, 44], as well as the successful experience in primary central nervous system lymphoma [13] and a potential benefit in bone lesions reported in other osseous cancer [45]. We also altered the dose of MTX to achieve better response in central nervous system. There is a puzzle when applying new regimens on patients with rare, life-threatening disease like LCH, ethical issues have been taken into consideration, and the trial was approved by the Ethical Institutional Review Board of the Peking Union Medical College Hospital in accordance with the Declaration of Helsinki.

In our cohort, the overall response is 100%, with a complete response rate of 16.7% and a partial response rate of 83.3%, the estimated average EFS is 48.9 months, and the overall survival rate after a median follow-up of 44 months was 97.2%. Although the rate of Non-Active disease (NAD) was relatively lower, most patients showed stable status without clinical manifestations. The overall recurrence rate in our study is 27.8%, which is much lower than that from our previous retrospective study [8] when compared to the classic VP regimen or intensive CEVP regimen (the overall recurrence rate was 73.3, 71 and 78.6% in the CEVP and VP group respectively). Although this result is limited by the relatively short follow-up, but it is also quite inspiring and deserves longer follow-up. In other studies that put effort in exploring an effective regimen for adult LCH patients. Saven and his colleague [46] explored the single agent cladribine in treating LCH in 13 adult patients, and this single agent showed overall response rate of 75%, and tolerable toxicity with hematologic adverse effect as the main toxicity. And this agent was further retrospectively analyzed in a 7-case cohort [47] showing durable effect in 86% patients despite the small sample size. Enrico Derenzini and his colleague [48] explored the efficacy of MACOP-B regimen in 11 patients, the overall response rate was confirmed to be 100%, with a complete response of 73% and a partial response rate of 27%, and overall progression free survival was 64% in this 11-case cohort. A brief summary of these regimen is shown in Table 4.

**Table 4:** Summary of trials exploring regiments treated for adult LCH patients

Year	Author	patient number	Fisrt line regimen	Overall response rate	PFS or reactivation	3–4 grade hematological toxicity
1999	Alan Saven	13	Cladribine	75%	N/A	Neutropenia 7/13 (53.8%)
2012	Maria A. Cantu	19	Vinblastine+ prednisone	16%	N/A	75%
		22	2-CdA	41%	N/A	37%
		24	ARA-C	79%	N/A	20%
2013	Z. ADAM	7	Cladribine	86%	N/A	Neutropenia 6/7 (86%)
2015	Enrico Derenzini	11	MACOP-B	100%	Overall 64%	Neutropenia 4/11 (36.4%)
2016	Duan MH	31	CEVP	68.8%	Reactivation rate 73.3%	Neutropenia 35.6% Thrombocytopenia 8.9%
		14	VP	70%	Reactivation rate 71%	Neutropenia 48.4% Thrombocytopenia 12.9%

*N/A* Not Applicable, *MACOP-B* cyclophosphamide, doxorubicin, methotrexate, vincristine, bleomycin, prednisone, *CEVP* cyclophosphamide, vindesine, etoposide, prednisone, *VP* vindesine, prednisone

The overall osseous regression rate was 100% in this reported cohorts, bone aching disappeared in most patients, and significantly improved in others. The abnormity on imaging scanning also improved with the complete resolution rate of 38.7% and partial response rates of 61.3%. The osseous regression is better when compared to the single agent Ara-C reported in the literature [10]. The CNS regression was also favorable on imaging scanning, with the complete response of 52% and partial response of 48%. although most patients manifested with diabetes insipidus as sequelae, but this can be well-controlled with drugs.

It is important to determine the prognostic factors timely to make clinical decisions. In the pediatric cohort, the age less than 2 years old, risk organ involvement, the duration of treatment, as well as the rapid response at 6 weeks all show clear prognostic value [2, 3, 6, 49, 50]. In the current cohort, we examined the value of risk organ involvement in multi-system LCH patients, despite the limited risk organ involvement sample and the limited recurrence cases, risk organ involvement still showed strong prognostic value in EFS, the estimated average EFS in MS-RO+ group was significantly lower than that in MS-RO- group (*p* = 0.001). This result confirms the prognostic value of risk organ involvement, and coincides with the result in pediatric group.

In addition to the high response rate, the MA regimen was also well tolerated. During the chemotherapy and follow-up, the main adverse effect is hematological toxicity, which is similar the study using MACOP-B regimen (Methotrexate, Doxorubicin, Cyclophosphamide, Vincristine, Bleomycin, Prednisone) [48]. A total of 80.6% patient encountered with neutropenia at different grade, and grade 4 neutropenia is the majority, but only 2 cases of grade 3 infection events were recorded, most patients recovered from neutropenia after supportive therapy including granulocyte colony-stimulating factor (G-CSF) infusion. Thrombocytopenia is one other common adverse effect in MA regimen [51, 52], the total incidence of thrombocytopenia was 41.7%, and the grade 4 event rate was 19.4%, although there is no life-threatening hemorrhage event recorded during the trial, we also recommend a conventional application of platelet infusion when considering the high prevalence of thrombocytopenia. Other non-hematologic adverse effect includes allergic reaction, liver function abnormity, and gastrointestinal events such as diarrhea, nausea, and vomiting, and all these adverse effects are well controlled and tolerated.

Prominent limitations of this study include insufficient sample number and the limited follow-up, especially the sample number of patients with risk organ involvement. Relative lower incidence of LCH in adult patients when compared to pediatric patients contributes to the limited sample number. However, considering the limited data in adult LCH patients, the current trial still provides some important and inspiring information for newly diagnosed adult patients. The combination of methotrexate and cytosine arabinoside showed favorable response in adult LCH patients, especially for patients with osseous lesions or central nervous system involvement. The prognostic value of risk organ involvement was confirmed by the inferior EFS duration in RO+ group when compared to patients without risk organ involvement. In addition, the regimen is safe for adult patients because tolerable adverse effects were observed during the follow-up. Obviously, a longer follow-up is needed for better understanding of the long-term survival, recurrence, and mortality of the MA regimen. Also, more evidence, especially evidence from large-scale clinical trial, is needed for the wider promotion and application of this regimen. International cooperation is highly desired for designing a randomized trial exploring the further efficacy of MA regimen in newly diagnosed adult LCH patient.

## Conclusion

For the moment, we recommend that MA may be a good choice for newly diagnosed adult LCH patients, and conventional application of platelet infusion for preventing or treating thrombocytopenia.

## Supplementary information

**Additional file 1: Figure S1.** The survival analysis of other predetermined subgroups.

## Data Availability

The datasets generated and/or analysed during the current study are not publicly available due to privacy of patients, but are available from the corresponding author on reasonable request.

## Abbreviations

AD: Active disease
CEVP: Cyclophosphamide, etoposide, vindesine and prednisone
CNS: Central nervous system
CTCAE: Common Terminology Criteria for Adverse Events
DFHR: Dihydrofolate reductase
EFS: Event-free survival
G-CSF: Granulocyte colony-stimulating factor
GR/PR: Good response or partial response rate
HS: Histocyte Society
JLSG: Japan LCH Study Group
LCH: Langerhans Cell Histiocytosis
MA: Methotrexate and cytosine arabinoside
MACOP-B: Methotrexate, Doxorubicin, Cyclophosphamide, Vincristine, Bleomycin, Prednisone
MAPK: Mitogen-activated protein kinase
MRI: Magnetic resonance imaging
MS: Multiple System disease
MS-RO+: Multi-system at risk disease
MS-RO-: Multi-system low-risk disease
MTX: Methotrexate
NAD: Non-active disease
ORR: Overall response rate
PCNSL: Primary central nervous system lymphoma
SS-m: Multifocal single-system disease
VP: Vindesine and prednisone

## References

[1] Gadner H, Grois N, Arico M (2001). A randomized trial of treatment for multisystem Langerhans’ cell histiocytosis. J Pediatr.

[2] Gadner H, Grois N, Potschger U (2008). Improved outcome in multisystem Langerhans cell histiocytosis is associated with therapy intensification. Blood..

[3] Gadner H, Minkov M, Grois N (2013). LCH-III therapy prolongation improves outcome in multisystem Langerhans cell histiocytosis. Blood..

[4] Gadner H, Heitger A, Grois N, Gatterer-Menz l, Ladisch S (1994). Treatment strategy for disseminated Langerhans cell Histiocytosis. Med Pediatr Oncol.

[5] Minkov M, Grois N, Heitger A, Potschger U, Westermeier T, Gadner H (2000). Treatment of multisystem Langerhans cell histiocytosis. Results of the DAL-HX 83 and DAL-HX 90 studies. DAL-HX study group. Klin Padiatr.

[6] Allen CE, Ladisch S, McClain KL (2015). How I treat Langerhans cell histiocytosis. Blood..

[7] Girschikofsky M, Arico M, Castillo D (2013). Management of adult patients with Langerhans cell histiocytosis_recommendations from an expert panel on behalf of euro-Histio-net. Orphanet J Rare Dis.

[8] Duan MH, Han X, Li J (2016). Comparison of vindesine and prednisone and cyclophosphamide, etoposide, vindesine, and prednisone as first-line treatment for adult Langerhans cell histiocytosis: a single-center retrospective study. Leuk Res.

[9] Tin SNW, Martinduverneuil N, Idbaih A (2011). Efficacy of vinblastine in central nervous system Langerhans cell histiocytosis: a nationwide retrospective study. Orphanet Jof Rare Dis.

[10] Cantu MA, Lupo PJ, Bilgi M, Hicks MJ, Allen CE, McClain KL (2012). Optimal therapy for adults with Langerhans cell histiocytosis bone lesions. PLoS One.

[11] Novotny L, Rauko P (2009). Cytarabine conjugates with biologically active molecules and their potential anticancer activity. Neoplasma..

[12] Garcia-Cano J, Roche O, Cimas FJ (2016). p38MAPK and chemotherapy: we always need to hear both sides of the story. Front Cell Dev Biol.

[13] Han X, Ji Y, Ouyang M, Zhu T, Zhou D (2017). Efficacy and safety of HD-MTX based systemic chemotherapy regimens: retrospective study of induction therapy for primary central nervous system lymphoma in Chinese. Sci Rep.

[14] Citterio G, Reni M, Gatta G, Ferreri AJM (2017). Primary central nervous system lymphoma. Crit Rev Oncol Hematol.

[15] Han CH, Batchelor TT (2017). Diagnosis and management of primary central nervous system lymphoma. Cancer..

[16] Moulignier A, Lamirel C, Picard H (2017). Long-term AIDS-related PCNSL outcomes with HD-MTX and combined antiretroviral therapy. Neurology..

[17] Bertino JR (2009). Cancer research: from folate antagonism to molecular targets. Best Pract Res Clin Haematol.

[18] Kantarjian HM, O’Brien S, Smith TL (2000). Results of treatment with hyper-CVAD, a dose-intensive regimen, in adult acute lymphocytic leukemia. J Clin Oncol.

[19] Windebank K, Nanduri V (2009). Langerhans cell histiocytosis. Arch Dis Child.

[20] Lahey ME (1981). Prognostic factors in histiocytosis X. Am J Pediatr Hematol Oncol.

[21] Howard SC, McCormick J, Pui CH, Buddington RK, Harvey RD (2016). Preventing and managing toxicities of high-dose methotrexate. Oncologist..

[22] Broadbent V, Gadner H (1998). Current therapy for Langerhans cell histiocytosis. Hematol Oncol Clin North Am.

[23] Eisenhauer EA, Therasse P, Bogaerts J (2009). New response evaluation criteria in solid tumours: revised RECIST guideline (version 1.1). Eur J Cancer (Oxford, England: 1990).

[24] Wen PY, Macdonald DR, Reardon DA (2010). Updated response assessment criteria for high-grade gliomas response assessment in neuro-oncology working group. J Clin Oncol.

[25] Berres ML, Lim KP, Peters T (2014). BRAF-V600E expression in precursor versus differentiated dendritic cells defines clinically distinct LCH risk groups. J Exp Med.

[26] Margo CE, Goldman DR (2008). Langerhans cell histiocytosis. Surv Ophthalmol.

[27] Malpas JS, Norton AJ (1996). Langerhans cell Histiocytosis in the adult. Med Pediatr Oncol.

[28] Maia RC, de Rezende LM, Robaina M, Apa A, Klumb CE (2015). Langerhans cell histiocytosis: differences and similarities in long-term outcome of paediatric and adult patients at a single institutional Centre. Hematology..

[29] Grana N (2014). Langerhans cell histiocytosis. Cancer Control.

[30] Monsereenusorn C, Rodriguez-Galindo C (2015). Clinical characteristics and treatment of Langerhans cell Histiocytosis. Hematol Oncol Clin North Am.

[31] Imashuku S, Shioda Y, Kobayashi R (2008). Neurodegenerative central nervous system disease as late sequelae of Langerhans cell histiocytosis. Report from the Japan LCH study group. Haematologica..

[32] Delprat C, Arico M (2014). Blood spotlight on Langerhans cell histiocytosis. Blood..

[33] Badalian-Very G, Vergilio JA, Degar BA, Rodriguez-Galindo C, Rollins BJ (2012). Recent advances in the understanding of Langerhans cell histiocytosis. Br J Haematol.

[34] Sahm F, Capper D, Preusser M (2012). BRAFV600E mutant protein is expressed in cells of variable maturation in Langerhans cell histiocytosis. Blood..

[35] Brown NA, Furtado LV, Betz BL, Noah A (2014). Brown, high prevalence of somatic MAP2K1 mutations in BRAF V600E–negative Langerhans cell histiocytosis. Blood..

[36] Chakraborty R, Hampton OA, Shen X (2014). Mutually exclusive recurrent somatic mutations in MAP2K1 and BRAF support a central role for ERK activation in LCH pathogenesis. J Am Soc Hematol.

[37] Chakraborty R, Burke TM, Hampton OA (2016). Alternative genetic mechanisms of BRAF activation in Langerhans cell histiocytosis. Blood..

[38] Badalian-Very G, Vergilio JA, Degar BA (2010). Recurrent BRAF mutations in Langerhans cell histiocytosis. Blood..

[39] Roden AC, Hu X, Kip S (2014). BRAF V600E expression in Langerhans cell Histiocytosis clinical and Immunohistochemical study on 25 pulmonary and 54 Extrapulmonary cases. Am J Surg Pathol.

[40] Ogawa M, Kobayashi M, Jimbo K (2016). Clinical profile and BRAF status of 30 Japanese patients with adult Langerhans cell Histiocytosis. Blood..

[41] Huang H, Lu T, Sun Y (2019). Association between clinicopathologic characteristics and BRAF (V600E) expression in Chinese patients with Langerhans cell histiocytosis. Thorac Cancer.

[42] Morimoto A, Ikushima S, Kinugawa N (2006). Improved outcome in the treatment of pediatric multifocal Langerhans cell histiocytosis: results from the Japan Langerhans cell Histiocytosis study Group-96 protocol study. Cancer..

[43] Morimoto A, Shioda Y, Imamura T (2016). Intensified and prolonged therapy comprising cytarabine, vincristine and prednisolone improves outcome in patients with multisystem Langerhans cell histiocytosis: results of the Japan Langerhans cell Histiocytosis study Group-02 protocol study. Int J Hematol.

[44] Citterio G, Reni M, Ferreri AJ (2015). Present and future treatment options for primary CNS lymphoma. Expert Opin Pharmacother.

[45] Harrison DJ, Schwartz CL (2017). Osteogenic sarcoma: systemic chemotherapy options for localized disease. Curr Treat Options in Oncol.

[46] Saven A, Burian C (1999). Cladribine activity in adult Langerhans-cell Histiocytosis. Blood..

[47] Adam Z, Szturz P, Vanicek J (2013). Cladribine (2-chlorodeoxyadenosine) in frontline chemotherapy for adult Langerhans cell histiocytosis: a single-center study of seven cases. Acta Oncol.

[48] Derenzini E, Stefoni V, Pellegrini C (2015). High efficacy of the MACOP-B regimen in the treatment of adult Langerhans cell histiocytosis, a 20 year experience. BMC Cancer.

[49] Katewa S, Sachdeva A, Dinand V, Yadav SP (2008). Impact of risk organ involvement on outcome in Langerhans cell Histiocytosis (LCH). Blood..

[50] Stine KC (2015). Risk organ + LCH gets the one-two punch?. Blood..

[51] Duan MH, Zhang Y, Zhang M (2016). Efficacy and safety analysis of the combination of cladribine, cytarabine, granulocyte colonystimulating factor (CLAG) regime in patients with refractory or relapsed acute myeloid leukemia. Zhonghua Xue Ye Xue Za Zhi.

[52] Umino K, Fujiwara S, Sato K (2017). High-dose methotrexate and Cytarabine-based multi-agent chemotherapy (modified Bonn protocol) for systemic lymphoma with CNS involvement. Acta Haematol.

