# Supplementary material for: The combination of methotrexate and cytosine arabinoside in newly diagnosed adult Langerhans cell histiocytosis: a prospective phase II interventional clinical trial
Source: BMC Cancer. 2020 May 18;20:433. doi: 10.1186/s12885-020-06872-8 (PMC7236107; doi:10.1186/s12885-020-06872-8)
Supplement: Supplementary file 1 — Additional file 1: Figure S1. The survival analysis of other predetermined subgroups. [file 12885_2020_6872_MOESM1_ESM.docx]

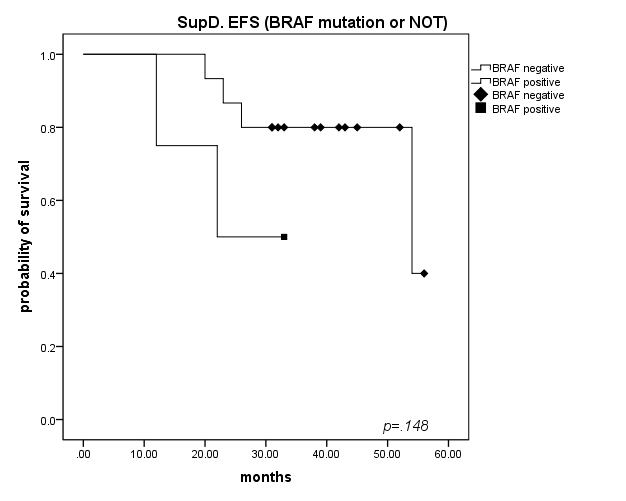

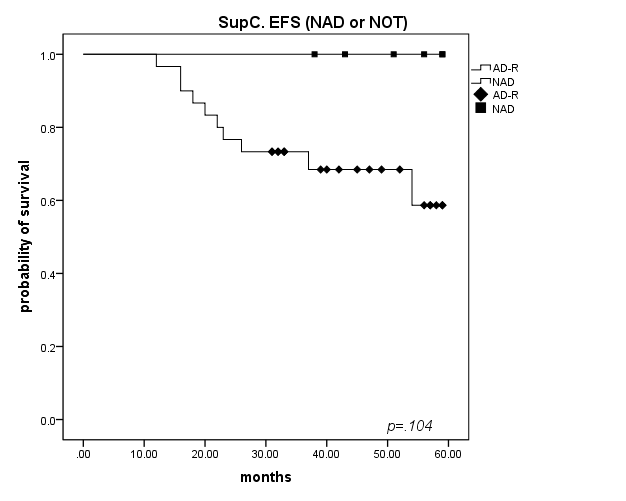

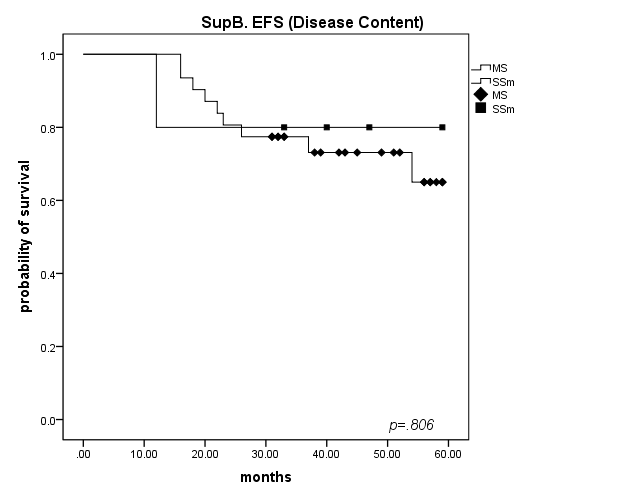

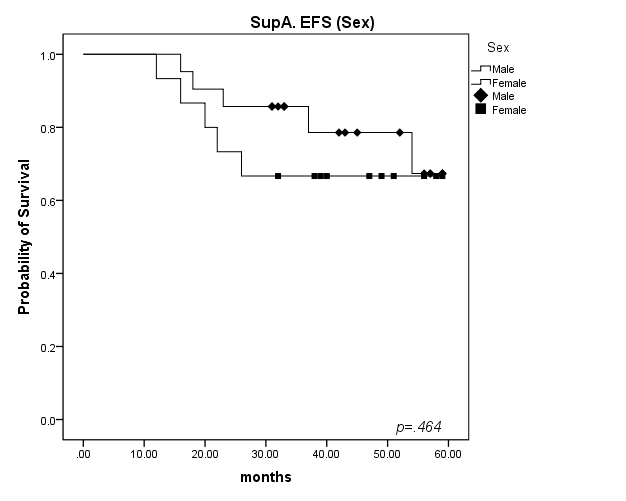


Supplementary Figure.1 Event free survival curve in proposed risk group. (A) EFS in male and female adult LCH patients treated with MA regimen in this trial. (B) EFS in adult LCH patients with single system disease and multisystem disease treated with MA regimen in this trial. (C) EFS in adult patients who achieved non-active disease and not after last cycle of MA regimen chemotherapy. (D) EFS in adult patients with LCH with positive and negative *BRAFV600E* mutation.
